# Hormonal regulation of telomerase activity and *hTERT* expression in steroid-regulated tissues and cancer

**DOI:** 10.1186/s12935-022-02678-9

**Published:** 2022-08-16

**Authors:** Mohammad Taheri, Soudeh Ghafouri-Fard, Sajad Najafi, Julia Kallenbach, Elmira Keramatfar, Golnaz Atri Roozbahani, Mehdi Heidari Horestani, Bashdar Mahmud Hussen, Aria Baniahmad

**Affiliations:** 1 Urology and Nephrology Research Center, Shahid Beheshti University of Medical Sciences, Tehran, Iran; 2 Institute of Human Genetics, Jena University Hospital, 07740 Jena, Germany; 3 Department of Medical Genetics, School of Medicine, Shahid Beheshti University of Medical Sciences, Tehran, Iran; 4 Department of Medical Biotechnology, School of Advanced Technologies in Medicine, Shahid Beheshti University of Medical Sciences, Tehran, Iran; 5 Department of Pharmacognosy, College of Pharmacy, Hawler Medical University, Kurdistan Region, Erbil, Iraq; 6 Center of Research and Strategic Studies, Lebanese French University, Erbil, Kurdistan Region Iraq

**Keywords:** Telomerase, Hormone, Growth factor, Prostate cancer, Breast cancer

## Abstract

Naturally, in somatic cells chromosome ends (telomeres) shorten during each cell division. This process ensures to limit proliferation of somatic cells to avoid malignant proliferation; however, it leads to proliferative senescence. Telomerase contains the reverse transcriptase TERT, which together with the *TERC* component, is responsible for protection of genome integrity by preventing shortening of telomeres through adding repetitive sequences. In addition, telomerase has non-telomeric function and supports growth factor independent growth. Unlike somatic cells, telomerase is detectable in stem cells, germ line cells, and cancer cells to support self-renewal and expansion. Elevated telomerase activity is reported in almost all of human cancers. Increased expression of *hTERT* gene or its reactivation is required for limitless cellular proliferation in immortal malignant cells. In hormonally regulated tissues as well as in prostate, breast and endometrial cancers, telomerase activity and *hTERT* expression are under control of steroid sex hormones and growth factors. Also, a number of hormones and growth factors are known to play a role in the carcinogenesis via regulation of *hTERT* levels or telomerase activity. Understanding the role of hormones in interaction with telomerase may help finding therapeutical targets for anticancer strategies. In this review, we outline the roles and functions of several steroid hormones and growth factors in telomerase regulation, particularly in hormone regulated cancers such as prostate, breast and endometrial cancer.

## Introduction

According to the Hayflick limit, most of somatic human cells divide naturally up to 40–60 times and eventually stop through cell cycle arrest [1]. Loss of regulation of cell division can lead to cancer development. Telomeres at the ends of chromosomes constitute repetitive sequences, which are required for maintenance of genomic integrity in proliferating cells by protecting the chromosomes against DNA damage. In human somatic cells, telomeres have a length of 7–17 kb which is required for their proper function and inhibition of DNA damage responses [2, 3]. In these cells, incomplete polymerase replication due to inability of the replicating lagging strand in synthesis of the chromosome extreme 3ˊend and nuclease processing leads to erosion of the telomere sequence by about 50–100 bp in each cell division [4, 5]. This phenomenon has been known to be associated with the cellular senescence [6].

Telomerase is a eukaryotic ribonucleoprotein complex [7]. This complex has six different subunits, namely heat shock protein 90, human telomerase RNA component (hTERC), dyskerin, telomerase-associated protein 1 (TEP1), p23, and human telomerase reverse transcriptase (hTERT) [8]. Telomerase complex is responsible for the genome maintenance by stabilizing the telomere length through adding repetitive sequences (5′-TTAGGG_*n*_ hexameric repeats in mammals) to telomeres during cell division [9]. Naturally, the telomerase-encoding gene, *hTERT*, shows a silent status in human somatic cells post birth, while stem cells, germ line cells, and malignant cells express significantly higher levels of *hTERT* expression [10]. Ectopic hTERT expression is known to bypass cellular senescence leading to extended replicative lifespan in the normal cells [11]. In malignancies, telomerase activity is required for unlimited replication of proliferating cells [12]. In a considerable proportion of 90% of human cancers, including breast and prostate malignancies, telomerase activation has been reported [13]. In a minority of malignancies a mechanism termed “alternative lengthening of telomeres” relying on the homology-directed DNA recombination (HDR) acts in a telomerase-independent manner to ensure the replicative potential of cancer cells [14]. However, in the majority of human cancers the main mechanism is through telomerase activation or upregulation [15]. In breast cancer, more than 90% of the malignant cases express high levels of *hTERT* [16]. Meanwhile, telomerase activity has been reported in 53 to 86% of breast cancer tissues [17, 18]. This telomerase activity has been found to be associated with tumor aggressiveness including high tumor size, advanced stage and lymph node metastasis [19]. Activity of this enzyme is decreased in the chemotherapy-treated tumors [19]. Most notably, telomerase activity is associated with decreased survival in the breast cancer patients [20].

Although telomerase is known for its critical telomere elongation function; its roles are not restricted to telomere homoeostasis. TERT also regulates a wide variety of important cellular functions such as gene expression, signal transduction, mitochondrial function and response to oxidative stress, and cell growth [7, 21]. These effects are called non-canonical functions of TERT, which have been reported not only in mammals, but also in other animals [22]. Importantly, enhanced telomerase activity in cancers has been shown to not only protect against telomere shortening, but also promote apoptosis resistance as another hallmark of cancer [23]. In HeLa cells, this effect is believed to be elicited via inhibiting the generation of radical oxygen species (ROS) through increasing the reduced Glutathione (GSH) and non-oxidized peroxiredoxin [24]. In addition, TERT has a role in improvement of mitochondrial function, since its over-expression has been associated with protection of mtDNA, increase in mitochondrial membrane potential and decrease in mitochondrial superoxide production and cell peroxide levels [25, 26]. Importantly, non-canonical functions of hTERT have been associated with oncogenic potentials consistent with its elevated telomeric function in cancer cells. Regulation of signaling pathways such as Wnt/β-catenin pathway, and oncogenic factors such as NF-κB, and Vascular endothelial growth factor (VEGF) are among the mechanisms through which telomerase contributes to cancer progression [7]. Moreover, the regulatory role of mitochondrial telomerase can lead to enhanced oncogenesis through the glycolysis metabolic pathway, and regulation of vasodilation [23].

The confirmed role of telomerase in various human cancers has suggested that hTERT inhibitors or anti hTERT immunotherapeutic approaches can be used as promising anticancer strategies [27]. Notably, a number of these strategies are being used into clinical trials [28, 29]. However, hTERT inhibitors should not only inhibit telomerase enzymatic activity but also should reduce its protein level because hTERT is involved in many other non-canonical pathways.

Protection mechanisms and the types of regulation of telomerase are being recognized during recent years. Most notably, substantial evidence proposes that hormones and growth factors have crucial roles in regulation of telomerase activity and expression of hTERT [30].

**Fig. 1:**
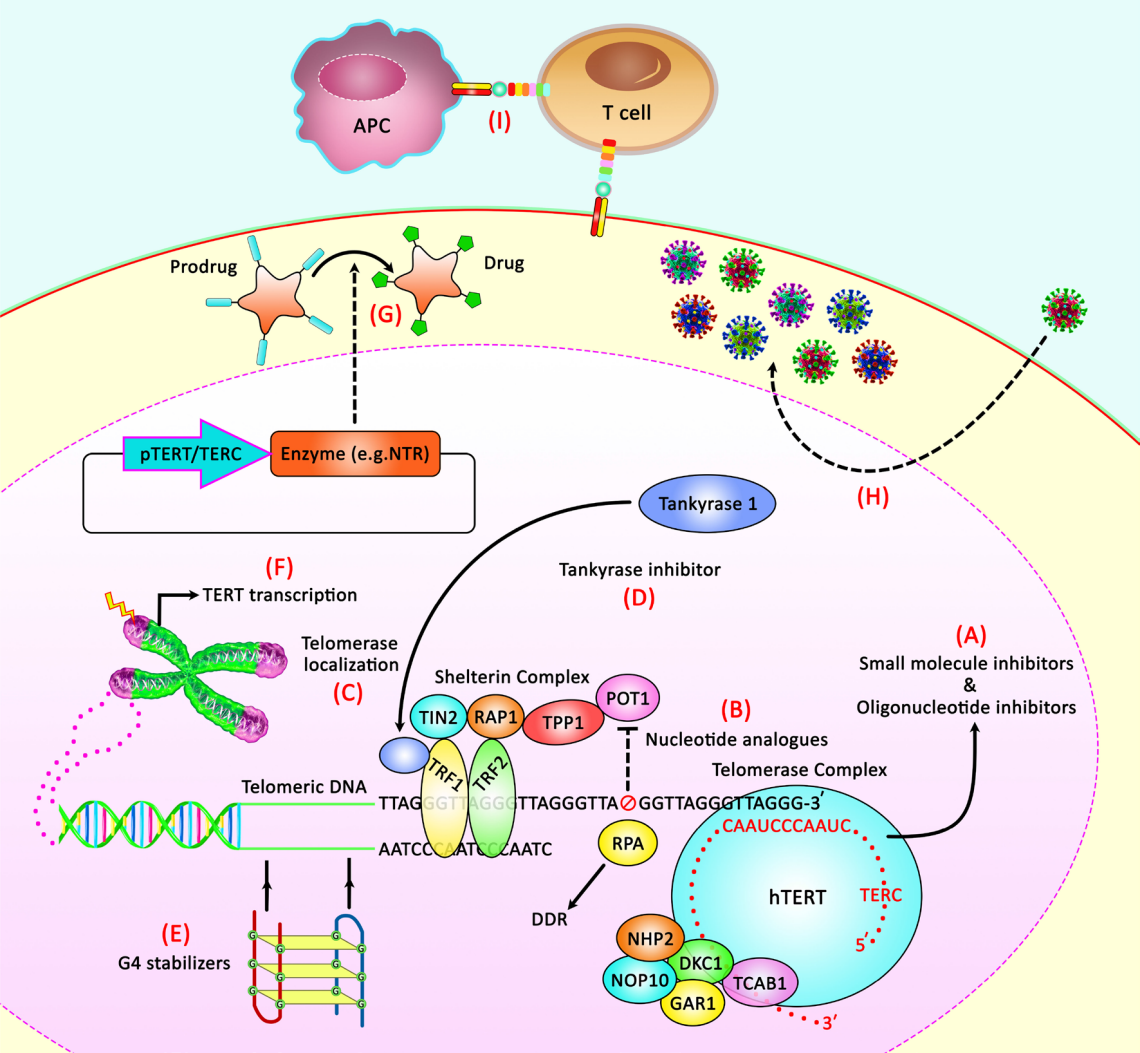
A schematic diagram of the telomerase remedial procedures. Various telomerase-based remedial strategies have been detected: **A** Suppressing telomerase via binding small molecules or oligonucleotides to TERT; **B** Weakening POT1 binding through attaching nucleoside analogues into lately created telomeres; **C** interfering with telomerase localization via Shelterin and TCAB1; **D** Attenuating the segregation of TRF1 from telomeres by Tankyrase suppressors; **E** Disrupting telomerase function via G-quadruplex stabilizers; **F** Targeting TERT transcription; **G** Transforming pro-drugs to active cytotoxic particles via gene therapy; **H** Specifically lysing tumor target cells via oncolytic viruses; **I** Stimulating immune responses against *hTERT* expressing tumor cells through telomerase peptide or DNA vaccines [31, 32]

Figure 1 shows different treatment modalities that target telomerase and telomeres.

## Telomerase and telomeres

To avoid cellular genome instability, genetic material in organisms should be protected against damage. Genetic information on linear chromosomes needs to be shielded from enzymatic attack. Furthermore, chromosome termini need to be hidden from natural DNA damage responses [33]. Telomeres are known as the eukaryotic genome protective elements located at the termini of linear chromosomes. Their importance was initially reported by Muller and McClintock [34, 35]. Initial studies by McClintock identified the fusion of broken chromosomes in the absence of telomeres [36]. The substantial role of telomeres in the protection of genome stability and cell functions depends on their structure composed of DNA and associated protein complexes bound to the repeat sequences [37]. These include particularly proteins involved in the DNA repair system through which telomeres are sheltered from DNA damage. This mechanism also provides the possibility of cell cycle-dependent regulation of chromosome termini [38]. Telomere DNA sequence is composed of GC-rich tandem repeats. This repeat in mammals is 5ˊ-TTAGGG-3ˊ, which can extend by hundreds of kilobases (10–15 kilobases long in humans at birth) [39]. Formation of large duplex t-loops at the ends of human chromosomes by telomeric repeat–binding factor 2 (TRF2) as a subunit in association with others including TRF2, and POT1 constitute the shelterin complex recognizing the repeat telomeric sequences and covers telomeres. This constitutes a mechanism to protect telomeres against nucleases and from telomeres being recognized as double strand DNA breaks [40, 41].

During each cell division in a majority of human and mouse cell types, telomeres shorten by a rate that varies depending on cell type and conditions and is between 50 and 150 bp in humans [39]. This issue results from incomplete replication of the telomeric lagging strand and can act as a mitotic clock preventing excess cellular proliferation occurring in cancer development [42]. It is also thought to play an essential role in the cellular senescence process limiting life span [43]. To address the replicative senescence problem due to the telomere erosion, several highly proliferating cell types including healthy stem cells and germ line cells, in addition to malignant cells, require activation of telomerase.

Initially named as terminal transferase, telomerase adds repetitive sequences to the 3ˊend of the telomeric DNA by an encoded reverse transcriptase from its intrinsic template RNA, while the complementary strand is synthesized through the normal replication process [44].

The length of telomere in humans is relatively short (5 to 15 kb) [45] which is shorter than mice (50 kb) [46], however humans have much longer life span compared with mice. In fact, the rate of telomere shortening instead of the initial telomere length is the important parameter that defines life span of species [47]. This rate is about 70 bp per year in human [45] and 7000 bp per year in mice [47].

## G-Quadruplex function in telomere

DNA sequences with consecutive guanine bases develop four-stranded G-Quadruplexes (G4) via Hoogsteen base-pairing. They are noncanonical secondary structures, have high thermodynamic stability and show a high rate of structural polymorphism [48]. Telomeres with G-rich repeats are among human genome sequences such as gene promoters, and DNA replication origins, which are able to form G4 structures with high conservation among divergent organisms like plants, yeast, and humans [49]. G4-Quadruplexes have been found in promoters with active transcription including that of the proximal *hTERT* promoter [50]. Telomeric G4 structures have been suggested to regulate (either positively, or negatively) telomerase, and protect telomeres as capping structures insulating them against DNA repair machinery and enhancing their maintenance via telomerase or recombination methods [49]. However, during telomeres extension, this structure is resolved in order to provide access by telomerase [49]. Telomerase has the ability of binding to and extending parallel G4 structures on telomeric overhangs but cannot act on antiparallel or hybrid quadruplexes. G4 structures play a role in lengthening telomeres by controlling *hTERT* expression and induction of genome instability in somatic cells [50]. As mentioned, this process is affected in cancer cells to support limitless division [51]. A number of ligands have been employed for quadruplexes as therapeutic targets [52]. About 1000 small molecules targeting quadruplexes have been known, among them some have shown anticancer properties [53]. These chemical ligands such as dimeric aryl-substituted imidazole (DIZ-3) have been revealed to act through stabilizing the telomeric G4 structures, and therefore, inhibiting cell proliferation in cancer cells [54]. Other telomerase inhibitors like tetra-(N-methyl-4-pyridyl) porphine (TMPyP4), berberine, and telomestatin inhibit telomere elongation via interacting with quadruplex DNA [55–57].

## Telomerase in development

In somatic cells, telomerase activity is generally reduced postnatally. While in embryonic stem cells telomerase is activated to maintain telomere length and immortality of these cells; this enzyme has low activity or even is absent in the majority of other types of stem cells irrespective of their proliferative ability. Therefore, even in stem cells, with the exception of embryonal stem cells and cancer stem cells, telomeres are shortened during replicative senescence, perhaps at a slower speed compared with normal somatic cells [3].

Mission of sperms to transfer intact genetic material to the next generation requires essential telomerase activity [58]. Oocytes in contrast to sperm cells do not possess telomerase activity which is only activated during later stages of development while initially telomeres are maintained by the ALT mechanism [59]. During different steps of development and organogenesis, telomerase activity is strictly regulated suggesting its substantial role in the organogenesis [60]. Telomerase activity is highly needed throughout the cell division for maturation and fertilization of gametes, and early development [61]. In human embryos, *hTERT* expression gradually decreases during development and fetal tissue differentiation and it falls early after gestation in several organs like heart and liver [62, 63]. Eventually, after birth, telomerase activity is not detected in most human somatic tissues. Overall, telomerase plays an essential role in fertilization and organ development by regulating cellular processes such as signaling pathways and apoptosis [64, 65] and its dysregulation can result in aging, as well as several human disorders such as dyskeratosis congenita [66] and idiopathic pulmonary fibrosis (IPF) [67].

## Telomerase in cancer

As a cancer hallmark, increased telomerase activity is reported almost in all human malignancies [68]. Increased *hTERT* expression or telomerase reactivation is required for limitless consecutive cellular proliferation of immortal malignant cells. Accordingly, mutations within the *hTERT* in the cancer cells have shown increasing effect on the telomerase activity [69]. These mutations have been found commonly in various human cancers. *hTERT* mutations are predominantly promoter mutations [70]. Notably, up to 90% of glioblastomas have been shown to have such mutations [71]. Also, several transcription factors with oncogenic features like c-MYC, NF-kB, and HIF-1 have been shown to up-regulate *hTERT*, while tumor suppressor factors such as p53, MZF-2 and SIP1 down-regulate *hTERT* [39]. Increased *hTERT* expressions along with frequent mutations in hTERT in the majority of human malignancies suggest a critical role for telomerase in enhancing cellular proliferation which is required for carcinogenesis. Accordingly, a number of clinical trials are being conducted to investigate the effect of telomerase inhibition using small molecules, oligonucleotides, or immunotherapy strategies as anti-cancer therapeutics [72].

As stated previously, telomerase effects are not restricted to chromosome ends and a body of evidence has shown non-canonical functions of hTERT independent of its characteristic telomere elongation features. Among them, some features have been associated with cancer cell capabilities to enhance their survival and metabolism. Although telomere maintenance is the most important function of telomerase in cancers, hTERT plays role in several other biological processes such as gene expression, signal transduction and coping with oxidative stress in mitochondria [73]. Moreover, it interplays with transcription factors playing role in inflammation and vascular genesis. All of these functions can consequently affect cell proliferation, migration, and regeneration, thus enhancing oncogenesis [7, 23]. Therefore, it is important to inhibit or inactivate the telomerase activity. To achieve this aim, we need to understand telomerase regulation in more detail, which can provide appropriate targets in cancer therapy. Among the regulators, we decide to focus on hormones that are well identified to regulate many biological functions.

In addition, the activity of telomerase is regulated by alternative splicing of hTERT mRNA. The pre-mRNA alternative splicing of hTERT at the post-transcriptional level is an important mechanism for modulation of telomerase activity. Alterations in splicing patterns happen in different stages of development, tumorigenic process, and in reaction to different stimuli. These alterations follow both tissue- and cell type-specific manner. Modulation of the type of hTERT pre-mRNA splicing is regarded as a novel strategy for treatment of cancer and aging-related disorders [74]. The *hTERT* gene has 16 exons and 15 introns. This gene can produce the full-length hTERT mRNA with an approximate size of 4.0 kb. At least seven selective splicing sites have been identified in the hTERT pre-mRNA. These sites include three deletion sites (α, β, and γ) and four insertion sites [75]. Any splicing happening in diverse arrangements of these sites will result in the production of a number of hTERT alternative splice variants at different levels. Yet, just the full-length hTERT mRNA with no deletion or insertion has the telomerase activity [76]. The 36 nucleotide deletion in exon 6 (α splicing site) eliminates the majority of the reverse transcriptase motif A. The β-deletion alternatively spliced variant is resulted from deletion of 182 nucleotides of exons 7 and 8. The deletion of 189 nucleotides in exon 11 (γ splicing site) eliminates regions that encode motifs D and E [77]. While the α- and γ-deletions are in-frame deletions, other deletions are out-of-frame [78]. Different functions have been attributed for alternatively spliced forms of hTERT. For instance, α-deletion and γ-deletion inhibit telomerase activity. β-deletion form inhibits telomerase activity but protects from apoptosis. Finally, Δ4–13 has extra-telomeric functions and stimulates cell proliferation [74].

hTERT alternative splicing variants can be used as prognostic or diagnostic markers in cancer patients. Notably, their expression has been correlated with histopathological and clinical variables. Expression of the β-deletion variant has been detected in stem and cancer cells. This variant has been found to be the hTERT transcript being most highly expressed in a panel of breast cancer cells. Splicing of this transcript has been shown to be regulated by a number of splice regulators, namely SRSF11, HNRNPH2 and HNRNPL. Forced over-expression of β-deletion protein leads to inhibition of endogenous telomerase activity. Up-regulated β-deletion protein has been demonstrated to reside the nucleus and mitochondria, protecting breast cancer cells from cisplatin-stimulated apoptosis [79].

Another study has shown that a splice site variant in *hTERT*, namely rs10069690 is a possible functional single nucleotide polymorphism since its T allele results in the co-production of full-length hTERT in addition to an alternatively spliced variant with inhibitory effects on telomerase activity. This inhibitory effect is more likely resulted from a dominant negative effect of the protein because telomerase exists as a dimer [80]. Most notably, this splicing variant of *hTERT* has been shown to interact with menopausal estrogen therapy to confer risk of ovarian cancer [81].

Moreover, regulatory T cells can inhibit proliferation of T and B lymphocytes as well as natural killer cells in a contact-independent process which involves activation of hTERT alternative splicing [82].

In addition, endonuclease G (EndoG) has been shown to induce splicing of hTERT and down-regulate telomerase activity [83]. Expressions of EndoG and hTERT splice variants have been found to be correlated in several colon cancer cell lines. Up-regulation of EndoG in a colon cancer cell line has led to downregulation of the active full-length hTERT variant and up-regulation of non-active alternatively spliced variants [83]. Nucleases can be induced by hormones, especially extra amounts of hormones. And it is highly possible that alternative splicing of hTERT can be induced by EndoG in response to hormones.

## Sex hormones and telomerase

### Estrogen and telomerase

Estrogen receptors (ERs) are members of nuclear hormone receptor superfamily and they are ligand-dependent transcription factors that modulate expression of a variety of genes [84, 85]. Estrogens mediate their effects through the ligand-dependent receptors (ERα and ERβ) and are essential for tissue development, growth and differentiation. Although estrogen withdrawal induces atrophic changes in many estrogen-dependent tissues, prolonged exposure to estrogen increases risk of some types of tumors like breast and ovarian cancers [86]. In fact, by interacting with one of their receptors, estrogens exert genomic and non-genomic biological effects which are well-characterized in various hormone-sensitive cancers such as breast, prostate, ovarian and endometrial cancer [85, 87]. Several lines of evidence suggest that estrogen controls telomerase activity directly and indirectly in estrogen target tissues [88–91] such as breast and endometrium [92–94] and prostate [95]. Estrogen deficiency leads to telomere shortening, while, its over-activity increases telomerase activity and maintains telomere length [86].

#### Breast cancer (BC)

The *hTERT* gene is a direct and indirect target of estrogen, which suggests the existence of hormone-dependent mechanisms to control telomerase activity [96, 97]. Noteworthy, there are two putative estrogen response elements (EREs) in the 5’ flanking sequence of *hTERT* promoter. The distal one is located at -2777/-2755 and the proximal one resides at −979/−956 [90]. Kyo et al. used the BC cell line (MCF-7) to examine the effect of estrogen on telomerase activity and observed that ER binds directly to the *hTERT* promoter via degenerated ERE motifs. In addition, c-Myc mediated estrogen-induced *hTERT* transcriptional activation suggests the presence of both direct and indirect ER activation pathways [96, 98].

#### Ovarian cancer

The estrogen-ER complex, by binding to the proximal ERE of *hTERT*, leads, to enhanced *hTERT* expression and telomerase activity in telomerase-negative primary ovary epithelial cells [99]. Treating Caov-3 human ovarian cancer cells with 17β-Estradiol (E2) induced telomerase activity after 6 h, reached a plateau after 24 h with declining thereafter. E2 not only increased hTERT protein but also *hTERT* mRNA level. In line with this, using ICI 182,780, a high affinity estrogen receptor antagonist, weakened E2-induced telomerase activity, which indicates an ER-dependent mechanism for regulating telomerase activity [100]. Interestingly, E2 induces phosphorylation of AKT, Src and PI3K. the E2-induced non-genomic AKT phosphorylation is reverted in the presence of ICI 182,780, of the Src inhibitor PP2, or the PI3K inhibitor (LY294002), which resulted in reduced E2-induced *hTERT* expression. Thus, this suggests that the Src-PI3K-AKT cascade is also involved in E2-induced *hTERT* expression [100]. The molecular basis might be that at posttranscriptional level AKT increases telomerase activity via phosphorylation of hTERT at Ser-824. Inhibiting estrogen-induced hTERT and AKT phosphorylation, and also estrogen-induced nuclear translocation of hTERT by LY294002 illustrates that phosphorylation of hTERT is an important pathway for both estrogen-induced hTERT nuclear translocation and telomerase activity [100].

#### Endometrial cancer

Estrogen regulates telomerase activity and *hTERT* gene expression in ER-positive (Ishikawa and ECC-1) and ER-negative (RL 95–2 and HEC-1B) endometrial cancer cell lines transiently transfected with ERα. This function of estrogen is exerted through direct interaction of activated ER with EREs in the *hTERT* promoter [90]. In addition, an E2-MAPK cascade was identified. Treating the Ishikawa cell line with E2 increased telomerase activity and *hTERT* expression in a dose and time dependent manner, while treating with the U0126 (a MEK inhibitor) inhibited E2-induced telomerase activity and also reduced *hTERT* expression. On the molecular level, E2 induces p44/42 MAPK phosphorylation and increases MAPK activity. On the other hand, treatment with U0126 blocked this phosphorylation. Thus, it appears that there is a crosstalk between E2 signaling and the MAPK pathway [91]. In line with this, luciferase assays illustrated that exposure to U0126 or ERK1/2-specific siRNA blocked the stimulatory effect of E2 treatment via EREs in the hTERT promoter. Thus, in endometrial cancer a crosstalk between E2 and MAPK pathway regulates telomerase activity [91].

#### Prostate cancer (PCa)

Increased telomerase activity is detected at the early stages of PCa [101, 102]. In the presence of E2 prostate epithelial cells (PrEC) show a rapid induction of *hTERT* expression within 3–6 h [103]. A mutation in the hTERT-ERE in PrEC abolished estrogen responsiveness. This observation highlights the importance and specificity of this motif. Specific and E2-dependent recruitment of ER_β_ and ER_α_ onto the hTERT promoter were illustrated in PCa cells using ChIP assays and accordingly hTERT expression was induced by E2 [103] indicating that in PCa the E2-ER-hTERT signaling is active. This result emphasizes the role of ER in regulation of telomerase activity that leads to potentially unlimited cell proliferation in PCa [103].

Taken together, E2 induces telomerase activity through *hTERT* at both transcriptional level and at post-translational level. This includes direct ER- ERE-dependent mechanism on chromatin and ER-Myc interaction as well as non-genomic E2-induced activation of MAPK-MEK and SRC-PI3K-AKT cascades, latter by AKT-dependent telomerase phosphorylation [100].

### Progesterone and telomerase

Substantial evidence insinuates that progesterone and growth factors including TGF-β have an inhibitory role in regulating telomerase activity and gene expression of *hTERT* [30].

#### Endometrial cancer

Recent studies show that although being somatic, the normal human endometrium expresses *hTERT*, and its regulation strongly depends on the menstrual phase, which indicates a regulatory role of progesterone in the regulation of telomerase activity [104]. High telomerase activity and short telomere length are the characteristics of proliferating endometrial epithelium cells in vivo and in vitro. Interestingly, the length of the telomerase varies based on the ovarian hormone cycle [105]. During the progesterone-dominant mid-secretory phase endometrial telomere lengths are remarkably shorter than in the proliferative phase. During the progesterone-dominant mid-secretory phase, the levels of endometrial telomerase activity, *hTERT* mRNA and protein as well as hTERC levels are reduced to their minimum levels. Accordingly, progesterone exposure inhibits telomerase activity in endometrial epithelium cells in vivo and decreases endometrial telomerase activity in explant and in vitro cultures in comparison with untreated cells [105]. Thus, in the progesterone dominant mid-secretory phase, shorter telomere length is observed, being in line with lowest telomerase activity, implicating progesterone negatively regulates telomerase activity and telomere length [105].

Endometrial cancer is initially, similar to the normal endometrium, a hormone-driven tissue [106]. Progesterone causes the transition of endometrial cells from the proliferative phase to the secretory phase [107]. The absence of progesterone receptors is one of the features of advanced endometrium cancer [108]. Notwithstanding that advanced and recurrent endometrial cancer can be treated with progesterone, and implementing progesterone may regress these tumors, it does not affect the survival of patients [109].

However, the *hTERT* promoter does not contain a canonical progesterone responsive element, hence classical PR-mediated direct effects are less probable [104, 110]. Interestingly, high telomerase activity, *hTERT* expression, and protein level that correspond to long telomere length in endometrium are rather characteristics of the ectopic secretory endometrium of endometriosis [105]. Importantly, resistance to progesterone causes the pathogenesis of endometriosis [110–112]. Thus, in the pathogenesis of endometriosis and progression of ectopic endometriotic lesions, the endogenous progesterone fails to inhibit telomerase and consequently may induce telomerase activity [110]. Thus, progesterone negatively regulates telomerase in the endometrium and antagonizes estrogen activity in female fertility organs [104].

#### Breast cancer (BC)

In breast tissues, progesterone operates in cooperation with estrogen to promote proliferative and pro-survival genetic programs [113]. It is demonstrated that the treatment of T47-D BC cells by progesterone downregulates the *hTERT* mRNA expression and telomerase activity. Moreover, this progesterone treatment is associated with a cell cycle blockage in the transition from G0/G1 to S-phase of the cell cycle [114]. Analyzing cells sorted by flow cytometry denotes that TA is lower in the G0/G1-phase compared to the S- or G2/M-phase, with or without hormone treatment. These results suggest that the *hTERT* gene is not a direct progesterone target, and the main reason for the down-regulation of telomerase activity after progesterone treatment is the cell cycle blockage and cell accumulation in the G0/G1-phase [114].

In a BC cell line, exposure to synthetic progestin, medroxyprogesterone acetate (MPA), suppresses mRNA expression of *hTERT*. This effect is similar to endometrial cancer and was even observed in the presence of estrogen [104, 114]. This treatment resulted in the cell cycle arrest in the late G1-phase being associated with inducing the cell cycle inhibitor p21 [104, 115].

More detailed investigation of hTERT regulation by progesterone implies that progesterone exhibits a time-dependent regulation on *hTERT* gene expression being adverse [104]. The effects of progesterone on *hTERT* gene expression have been analysed by culturing PR-positive T47-D human BC cells in the absence or presence of MPA at various concentrations. These findings indicate that progesterone substantially induces *hTERT* mRNA expression within a period of 3 h after treatment. This ephemeral effect reaches its peak about 12 h after exposure and subsequently decreases. Despite the transient effect of progesterone on *hTERT* gene expression in short exposure periods, hormone treatment for longer than 48 h antagonizes the estrogen impact and suppresses the estrogen-induced activation of *hTERT* expression [104].

Mechanistically, overexpression of p21 leads to the significant inhibition of *hTERT* mRNA expression. Furthermore, the progesterone inhibitory effects on *hTERT* expression are considerably attenuated in cells transfected with p21 expression vector [104].

In order to investigate the effect of p21 on *hTERT* expression, experiments with T47-D cells by p21 overexpression leads to suppression of *hTERT* expression. Use of overexpression and knockdown experiments suggest that the incubation extended to more than 48 h, progesterone promoted p21 levels, which in turn suppressed the expression of *hTERT*. Still, the detailed underling mechanism is not known how p21 suppresses *hTERT* expression by exposure to progesterone [116]. Further mechanistic details indicate that the mitogen-activated protein kinase (MAPK) signaling pathway mediates both the short- and long-term impacts on *hTERT* gene expression by progesterone. Moreover, the findings suggest that p21 plays a significant function in the long-term effect of progesterone on *hTERT* gene expression [104].

### Androgen signaling and regulation of telomerase

The development and growth of PCa is strongly controlled by androgens that mediate their effects through the androgen receptor (AR) [117]. Notably, AR is important to maintain telomere stability and is involved in the replication of telomere DNA in PCa cells. Inactivation of AR and androgen deprivation causes telomere dysfunction [118]. Further, it was shown that AR interacts with telomeric proteins of the shelterin complex such as TIN2 [119].

#### PCa

Telomerase plays also a pivotal role in PCa, which is the most commonly diagnosed non-cutaneous cancer and the second leading cause of cancer deaths in males in Western countries [120]. Importantly, studies indicated that telomerase reactivity increases with the tumor grade of PCa [121]. In PCa, *hTERT* expression positively correlates with clinicopathological features such as Gleason score, tumor differentiation, and serum levels of prostate-specific antigen (PSA), as the most commonly used biomarker in diagnosis of PCa, along with recurrence and worse survival in the affected patients [122, 123]. While highly elevated expression of *hTERT* in PCa tissues with poor differentiation or in cases with metastasis is seen, the normal prostate or benign prostate hyperplasia (BPH) tissues do not express *hTERT* at detectable levels [124]. Interestingly, reports indicate repression of telomerase in the normal prostate by androgens. Castration of animals is known to decrease androgen levels and results in elevated telomerase activity that is repressed by androgen treatment [101, 102]. This suggests that in PCa the androgen-mediated repression is override or abrogated. Indeed, in PCa cell lines with mutations at androgen receptor (AR) gene that have been known to positively regulate *hTERT* expression [125]. Accordingly, reduction of AR in cells treated with the anti-cancer agent methaneseleninic acid (a seleninic acid with the chemical formula CH_3_SeO_2_H) has been associated with reduced *hTERT* expression in both wild type and AR-mutated cells [126]. However, the use of supraphysiological androgen treatment used in the bipolar androgen therapy (BAT) indicates that high doses of androgens repress telomerase activity by transrepression of the *hTERT* expression in AR positive cells and in human PCa organoids [127]. This indicates that BAT has tumor suppressive activity and that the pathway and molecular mechanism of androgen-mediated repression of telomerase in PCa is still active, albeit at higher androgen doses.

However, androgen regulation of hTERT is controversial and more complex than originally expected. Both, activation and repression of hTERT by androgens have been described. Importantly, the first in vivo studies of telomerase in rats indicated that normal prostate glands lack telomerase activity. However upon castration, leading to androgen deprivation, telomerase activity was induced [95]. Moreover, similar results have been confirmed in rhesus monkey [128]. Treatment of animals with androgens reverted the induced telomerase activity indicating that androgens repress telomerase activity.

On one hand, androgens were reported to induce telomerase activity by recruitment of AR to the endogenous *hTERT* promoter in androgen-sensitive LNCaP cells. This effect was presumably indirect as an *hTERT* promoter construct was not activated by androgens in reporter assays [125, 129]. Deprivation of dihydrotestosterone (DHT) from cell culture medium of LNCaP cells, led to abrogation of the telomerase activity. However, in androgen-independent TSU-Pr1 or DU145 PCa cells DHT did not modulate the telomerase activity indicating that the AR mediates the androgenic response [130]. Nevertheless, a study of 30 PCa patients that received androgen deprivation therapy (ADT) before radical prostatectomy showed that ADT inhibits *hTERT* reactivation [131]. It supports the assumption that androgens activate telomerase function. Further evidence from xenografts and human PCa studies indicated that endogenous wild-type AR stimulates *hTERT* expression [131]. Suppression of AR signaling by AR knockdown using a doxycycline-inducible shRNA lentiviral system or treatment with AR antagonists such as bicalutamide decreased mRNA levels of hTERT and telomerase activity in LNCaP cells, confirming a positive regulation of hTERT/telomerase by the AR [129, 132]. Together, these investigations suggest a stimulatory effect of androgens via AR on *hTERT* expression in androgen-dependent PCa cells.

On the other hand, data suggest opposite regulation of telomerase in castration-resistant PCa (CRPC). It has been shown that exogenously overexpressed wild-type AR, but not the AR mutant T877A suppressed *hTERT* transcription by inhibition of the *hTERT* promotor activity in an AR null PCa cell line indicating that AR exhibits inhibitory effects on hTERT in these cells [133]. Further studies support the observation that AR-mediated signaling negatively regulates *hTERT* expression in CRPC [134]. It leads to the assumption that AR drives a distinct transcriptional program in CRPC cells.

A recent contribution shed light on the inconsistencies of androgenic stimuli on hTERT/telomerase activity by revealing a concentration-dependent effect of androgens on *hTERT* expression. Induction of *hTERT* expression was mainly observed by physiological, low androgen level (LAL), while high, supraphysiological androgen level (SAL) used in BAT suppressed hTERT. In line with this, SAL suppressed *hTERT* expression in ex vivo treated human PCa samples and cancer spheroids from CRPC cells [127]. The suppression of *hTERT* by SAL might explain in part the antitumor effects observed with the SAL treatment. Interestingly, it is suggested that the AR-induced transrepression of hTERT is mediated by the tumor suppressor proteins, inhibitor of growth 1 and 2, ING1 and ING2, respectively.

Interestingly, according to the biphasic actions of androgens, two responsive hTERT promoter regions were identified. A positive androgen response element (pARE) in the distal and a dominantly acting negative ARE (nARE) in the proximal promotor region of *hTERT* that mediates transrepression by androgens were identified by chromatin immunoprecipitation and reporter assays [127]. It is hypothesized that LAL activates the positive ARE at – 4 kb leading to upregulation of hTERT, whereas at SAL, the nARE predominates control of *hTERT* transcription resulting in androgen-mediated suppressed *hTERT* expression.

In conclusion, the differences in hTERT regulation by androgens might depend on the cell context, androgen concentration and treatment period. Furthermore, the underlying findings suggest that the mechanisms of androgen-mediated regulation of hTERT change during the progression of PCa.

In addition, also the type of androgen used in the experimental setup is relevant. It was shown that DHT metabolites can activate ERβ, which is recruited to the *hTERT* promoter resulting in the activation of *hTERT* expression [135–137]. Hence, it suggests that a high DHT metabolism in cells might activate *hTERT* expression instead of the expected repression. Thus, for these reasons, these above-mentioned factors should be taken into account for the analysis of androgenic regulation of telomerase activity and *hTERT* expression. In general, the androgenic regulation of hTERT requires to be further investigated in patients treated with hormone therapies, as it might help to evaluate hTERT as a diagnostic marker and to develop new antitelomerase therapies.

and physical stressors, the body releases hormones including glucocorticoids, catecholamine (Epinephrine, Norepinephrine), gonadotropin, growth hormone, and vasopressin.

**Stress hormones and telomerase**

In stressful situations such as work pressure, psychosocial and physical stressors, the body releases hormones including glucocorticoids, catecholamine (Epinephrine, Norepinephrine), gonadotropin, growth hormone, and vasopressin.

Interestingly, telomeres are not only affected by replication-induced telomere shortening, but also by stress. Deficiency of a telomere-specific damage repair in the stressful situation, cause telomere shortening [42, 138]. Also, stress of pregnant mothers causes shorter telomere length in offspring. An association has been reported between maternal exposure to severe psychosocial stress during pregnancy and shorter telomere length in offspring [139, 140].

Further, Choi and colleagues showed that exposure of human T lymphocytes to the glucocorticoid cortisol is associated with a significant reduction in telomerase activity [141]. Exposure to high concentrations of synthetic glucocorticoids (cortisol and dexamethasone) decreases expression and activity of telomerase in non-cancer cells like activated human T lymphocytes. This is mediated by inhibition of *hTERT* gene expression [141–144]. However, prolonged exposure to physiological concentrations of either cortisol or dexamethasone is not sufficient to accelerate telomere shortening in cultured human fibroblasts [145] since none of the two glucocorticoids (cortisol and Dexamethasone) significantly influenced telomere length in cultured fibroblast cells. In fact, normal human fibroblasts do not possess telomerase activity which might explain the missing effect of glucocorticoid treatment.

Although stress hormones are necessary and helpful to pass stressful situations but may have negative side-effects and can cause various diseases like cancer [146, 147] indicating an association between stressful life style and increased cancer occurrence.

### PCa

In patients with low-risk PCa, short telomere length in peripheral blood mononuclear cells (PBMCs) is in association with raised concentration of stress hormones like catecholamine and cortisol [148].

For appropriate telomere function, the shelterin complex comprised of several proteins like telomere repeat-binding factor 1 and 2 (TRF1, TRF2), protection of telomeres (POT1) and TRF1-interacting nuclear factor 2 (TIN2) are necessary to protect telomere degradation and control also telomere length. On one hand, POT1 binds to TRF1 by mediating of TIN2 in telomere region and on the other hand, binds to the TTAGGG repeats of telomere at 3' overhang. This complex prevents binding of telomerase to the ends of chromosome and avoids increase in telomere length outside the late S-phase [149, 150].

In normal prostate cells, compared to cancerous cells, a lower expression of GnRH-R was reported [151]. As a side note, another activator ligand that can bind and activate GnRH-R is a synthetic 16-amino acid fragment derived from hTERT protein that was developed to use for immunotherapy of many types of solid tumors [152]. This peptide fragment leads to anti-proliferative and pro-apoptotic effects on two androgen-dependent and CRPC PCa cell lines. Vaccination with this amino acid peptide may activate the immune system to mount cytotoxic T-lymphocyte response against *hTERT* expressing tumor cells.

### Breast cancer

Butler et al*.* in human breast tumor samples showed that activation of the glucocorticoid receptor in the presence of glucocorticoids like dexamethasone leads to potent upregulation of TRF1, TIN2 and POT1 proteins, which avoid binding of telomerase to the end of chromosomes [153].

### Ovarian cancer

In ovarian cancer two important factors that participate in telomerase regulation (hTERT) are HIF-1α and c-Myc. Norepinephrine leads to strong upregulation of HIF-1α and c-Myc genes and increases norepinephrine-induced *hTERT* expression. cAMP-dependent protein kinase A (PKA) is one of the underlying factors, which regulates both Norepinephrine-induced HIF-1α and c-Myc expression. This was confirmed by using the PKA inhibitor, H89, that inhibits HIF-1α and c-Myc expression and accordingly leading to *hTERT* repression [154]. The non-receptor tyrosine kinase Src is another important factor in norepinephrine-mediated increase in *hTERT* expression [155]. Src phosphorylation is a mediator in norepinephrine -dependent tumor metastasis in cancers especially of ovarian cancer. By using an inhibitor of Src in ovarian cancer cell lines (SKOV-3 and PA-1), norepinephrine-induced HIF-1α, c-Myc expression was inhibited leading to reduced *hTERT* expression. Thus, HIF-1α, c-Myc and Src stimulated through norepinephrine modulate *hTERT* expression in ovarian cancer to promote progression and invasion [154, 155]. In addition, norepinephrine and overexpression of *hTERT* lead to decreased expression of the epithelial marker E-cadherin and to an increase of both mesenchymal N-cadherin and Slug marker expression, which induce EMT as a critical step in cancer metastasis [154, 156].

### Endometrial cancer

In endometrial cancer, gonadotropin releasing hormone (GnRH) seems not to affect telomerase activity but can inhibit *hTERT* mRNA expression [157]. Telomerase activity in the human endometrium is activated by E2 during the proliferative phase of the menstrual cycle. However since GnRH can block steroid secretion via MAPK signaling, GnRH indirectly inhibits estrogen-induced telomerase activity in human endometrium cancer [158]. Since GnRH agonists inhibit sex steroid secretion, it is assumed that the absence of E2 stimulation is one of the mechanisms to inhibit telomerase activity in the human endometrium.

**Fig. 2:**
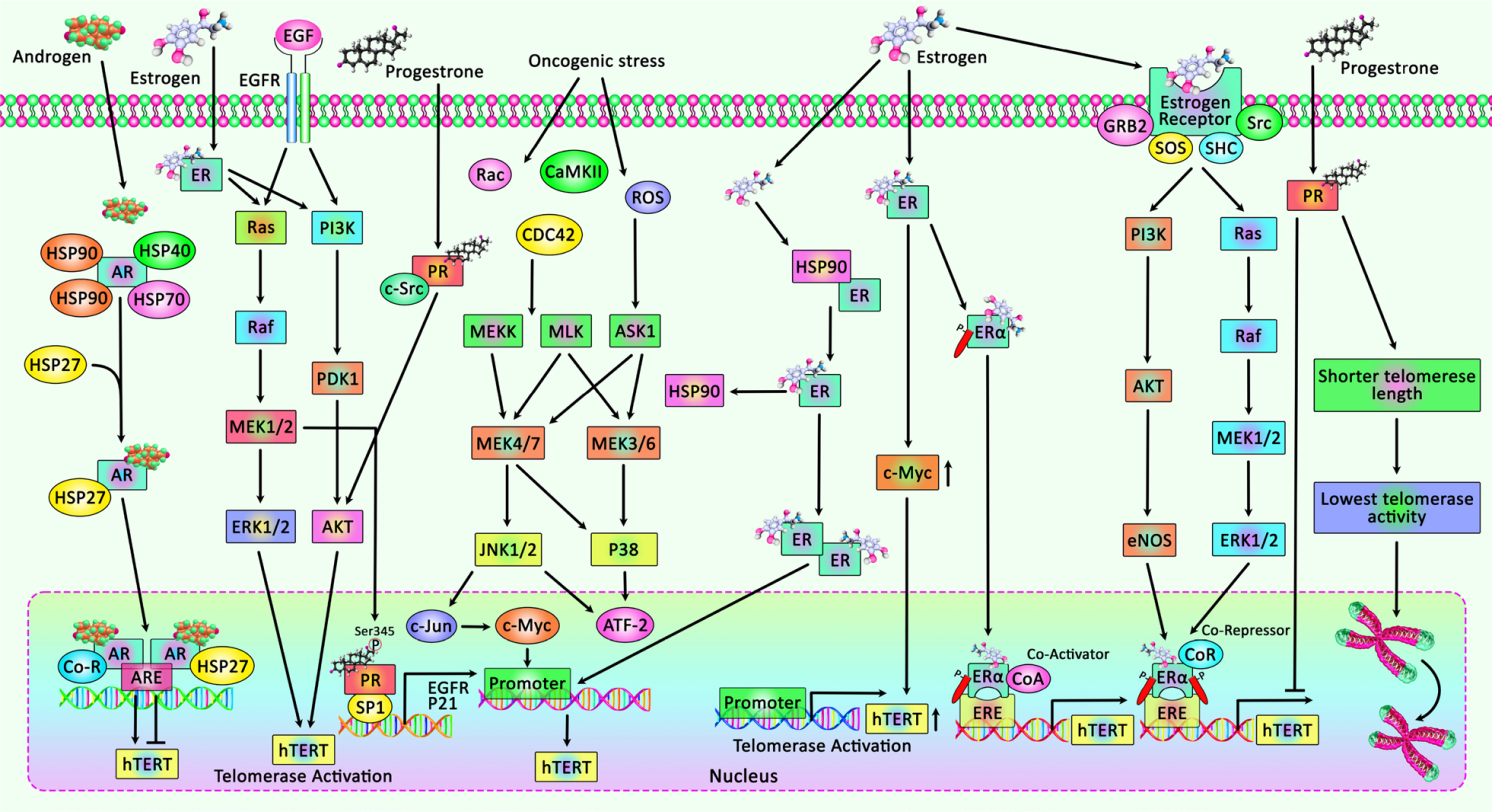
A schematic illustration of the mechanisms of hormonal modulation of telomerase in cancer cells. Accumulating findings have detected that androgen receptor, estrogen receptor, and progesterone receptor can play a crucial role in the regulation of hTERT expression in tumor cells. In addition, stress hormones can lead to overexpression of cellular telomerase activity in response to various environmental stimuli [159]

Figure 2 depicts the hormonal regulation of telomerase activity in tumor cells.

## Regulation of telomerase by growth factors and growth hormone receptors

A number of studies have also demonstrated the regulation of cell proliferation and division by growth factors. For instance, IGF-1 has been found to affect life span of mammals via controlling the cell division [160]. Importantly, growth factors have been demonstrated to exert this role through controlling the telomerase activity.

### Epidermal growth factor (EGF)

**Fig. 3:**
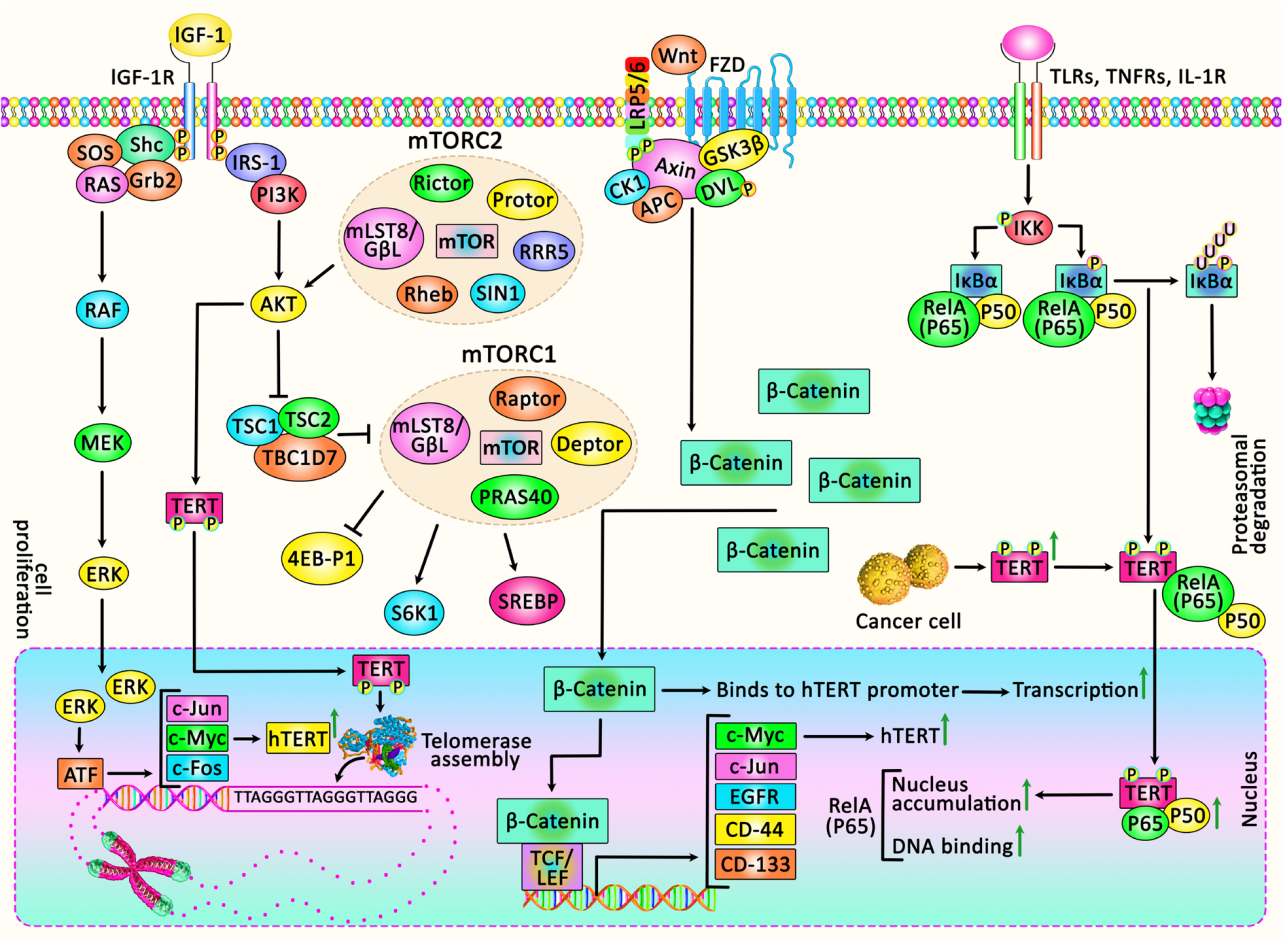
A schematic representation of the crosstalk between *hTERT* expression and various signaling pathways in cancer cells. Accumulating findings have demonstrated that telomerase could play a crucial role in unlimited proliferation and cellular immortality in various human tumor cells. Telomerase function could be modulated through several intracellular signaling cascades containing MAPK/ERK, PI3K/AKT/mTOR, Wnt/β-Catenin, and NFκB that mostly contribute to inflammation, EMT, cancer cell invasion, and metastasis [166]

Epidermal growth factor (EGF) controls proliferation, migration, and invasion through multiple signaling pathways in various cell types, while it does not affect normal cells like fibroblasts [30]. EGF binding to the receptor (EGFR) stimulates its intrinsic tyrosine kinase activity leading to receptor phosphorylation and induction of a number of signaling pathways. Several growth-associated genes such as *c-jun* and *c-fos* are also transcriptionally activated, through which EGF causes promotion of cellular proliferation [161]. MAPK/ERK signaling has been suggested to be involved in the effect of EGF on telomerase activity [162]. These pathways followed by EGF activation are known to promote carcinogenesis in a wide variety of human solid tumors like epithelial malignancies, in which activated EGF signaling particularly through various mutations in EGFR plays an essential role [163]. EGF has also been shown to positively affect telomerase activity as it is expected to be elevated in cancer cells. Treatment of EGFR-expressing cancer cells with EGF shows increased *hTERT* expression, while EGFR inhibition using AG 1478 shows the reverse effect [162, 164]. It can enhance the telomerase activity through overexpression of *hTERT*, which suggests transcriptional activation [162]. In BC patients, it has been reported that human epithelial growth factor 2 (HER2) and ER81 transcription factor can cause synergistic elevation of *hTERT* transcription [165]. Figure 3 represents the role of some intracellular signaling cascades in regulating *hTERT* expression in tumor cells.

### Fibroblast growth factor (FGF)

Fibroblast growth factors (FGFs) initially were identified as angiogenic factors; however, now they are considered as pleiotropic mitogens, show expression in a wide variety of cells. The signaling network demonstrates autocrine and paracrine activities, which play a role in several biological processes like embryogenesis, tissue development, tissue regeneration, and cancer development [167]. FGFs are particularly involved in tumor development and progression via regulation of cellular proliferation, migration, differentiation, and survival [168]. FGF-treated cells show elevated *hTERT* expression, increased telomere length, and also enhanced proliferation and expansion [169, 170]. Accordingly, FGF-mediated upregulation of *hTERT* extends lifespan and population doublings of embryonic stem cells [170]. FGF has also been recognized to restore telomerase activity in quiescent cultured human umbilical vein endothelial cells, but this effect was not seen for VEGF [171]. This effect is known to be exerted through elevating the *hTERT* gene transcription [172]. Wnt/β-catenin pathway is involved in the mechanism through which FGF upregulates hTERT [170].

## Transforming growth factor-β (TGF-β)

Transforming growth factor-β (TGF-β) shows both pro-proliferative activity in cancer cells and antiproliferative function in normal development acting as oncogene or tumor suppressor, respectively [173]. Mainly, potent anti-proliferative effects have been described for TGF-β in a wide variety of cell types including cancer cells [174]. Thus, TGF-β is considered as the only growth factor with profound inhibitory effects on cell growth [159]. These effects are believed to be conducted through overexpression of the cell cycle suppressors, repressing the cell cycle stimulators, and via signaling pathways like MAPK and PP2A [175]. Accordingly, TGF-β has shown to suppress telomerase activity, as its levels are negatively associated with telomerase activity [176], which is conducted via repression of *hTERT* transcription [177]. Mechanistically, it is suggested that TGF-β-stimulated Smad3, through inhibition of c-Myc, or *SIP1* are responsible for repressing the *hTERT* transcription [175, 178]. On the other hand, TGF-β is known to promote epithelial-mesenchymal transition (EMT) in cancer cells and hence enhance cancer progression [179]. In fact, TGF-β has paradoxical roles in different stages of cancer progression [180]. This cytokine regulates homeostasis and inhibits tumor progression directly via cell-autonomous tumor-suppressive effect (cytostasis, differentiation, apoptosis) or indirectly via affecting the stroma (inhibition of inflammatory responses and stroma-originated mitogens). But, once cancer cells become defective in TGF-β tumor-suppressive responses, they utilize TGF-β to their benefit to induce immune evasion, growth factor synthesis, conferring to an invasive phenotype with ability to metastasize [180].

### IGF-I

Insulin-like growth factor 1 (IGF-I) level increases during acute physical stress [181]. IGF and IGF-binding protein (IGFBP), which carries IGF to the IGF receptor, make a complex being involved in cellular growth. This axis has an important role in modulating human carcinogenesis like activation of telomerase activity. High telomerase activity was measured in response to IGF-I treatment in both androgen-dependent and androgen-independent PCa cell lines. Both IGF and IGFR activation lead to enhanced PI3-kinase-AKT pathway signaling to up-regulate *hTERT* expression and telomerase activity [182].

## Possible therapeutic applications of modulation of hormone-telomerase interactions in treatment of cancer

As previously stated, elevated telomerase expression and activity is a hallmark of almost all human cancers, while normal cells except for embryonic stem cells, and germ line cells lack telomerase expression [3].

In fact, telomerase reactivation or increasing *hTERT* expression acts as a mechanism to bypass the cellular senescence in malignant cells for achieving limitless proliferation. Due to frequent telomere shortening, telomerase inhibition has shown to cause increased apoptosis or senescence, and repression of cell proliferation leading to death of cancer cells prior to dissemination [183]. This makes telomerase a potential target for anti-cancer therapeutic strategies, particularly since telomerase can also contribute to drug resistance [184]. However, telomerase regulation is highly complex suggesting challenges to its targeting approaches [185]. Targeting telomerase itself using antisense oligonucleotides, ribozymes, peptide nucleic acids, and small molecules may help find a therapeutic solution for human cancers [186]. Hormones as regulators of telomerase activity and *hTERT* expression are also suggested as potential candidates of combined anti-cancer therapeutics in fighting against malignancies. This issue can possess high importance particularly in hormone-dependent cancers like PCa, BC and endometrial cancer. A successful experiment by Marconett et al. [187] demonstrated that the natural herbal product indole-3-carbinol (I3C) with antiproliferative properties represses *hTERT* expression through modulation of ERα and Sp1 transcription factor in BC cells. Overexpression of both ERα and Sp1 reversed the inhibitory effect of I3C on *hTERT* expression. I3C, also has been shown to inhibit cell proliferation in MCF-7 BC cells via disruption of IGFR-1 expression through ERα [188]. RNA interference-mediated HER2 silencing in another experiment has shown increased radiosensitivity via repressing *hTERT* expression in human BC cells [189]. Telomere homolog oligonucleotides or T-oligos with homologous sequences pair to the 3′ single-stranded overhang of the mammalian telomeres [190]. They have shown anticancer effects via telomerase inhibition, while in combination with EGFR inhibitor Gefitinib additively inhibits the cancer cell growth [191].

## Conclusion and future perspectives

The telomerase ribonucleoprotein complex is mainly responsible for adding repeat sequences to the ends of eukaryotic chromosomes. This function not only is involved in natural processes but also is mandatory for avoiding cellular senescence in a number of stem and cancer cells. Moreover, it plays role in embryonic development. Detectable expression of *hTERT* in more than 90% of the malignant cells confirms its role in cancer progression. A number of non-canonical functions have been identified for *hTERT*, including gene expression, signal transduction, mitochondrial function and response to oxidative stress, and cell growth, through which elevated *hTERT* expression in cancers is believed to enhance carcinogenesis. A number of sex hormones, androgens, and growth factors have been found to upregulate or to downregulate *hTERT* expression, so playing roles in the development and progression of cancers in androgen-responsive tissues like prostate, breast, and endometrium. Since telomerase activity is not required for somatic cells, the telomerase is a potential target for treatment of cancer. Theoretically, direct targeting of telomerase activity or targeting regulatory hormones by receptor agonists or antagonists as well as growth factors may help fight against enhanced *hTERT* expression or telomerase activity in cancer microenvironment. Further exploration of the precise mechanisms involved in the hormone-telomerase interactions not only increases our knowledge about the regulation of critical cellular processes, but also facilitates development of therapeutic modalities for human malignancies affected by hormones.

## Data Availability

The analyzed data sets generated during the study are available from the corresponding author on reasonable request.

## Abbreviations

hTERT: Telomerase reverse transcriptase
HDR: Homology-directed DNA recombination
VEGF: Vascular endothelial growth factor
TRF2: Telomeric repeat–binding factor 2
IVF: In vitro fertilization
PrEC: Prostate epithelial cells
MAPK: Mitogen-activated protein kinase
AR: Androgen receptor
PSA: Prostate-specific antigen
BPH: Benign prostate hyperplasia
BAT: Bipolar androgen therapy
DHT: Deprivation of dihydrotestosterone
ADT: Androgen deprivation therapy
CRPC: Castration-resistant PCa
LAL: Low androgen level
SAL: Supraphysiological androgen level
pARE: Positive androgen response element
PBMCs: Peripheral blood mononuclear cells
TRF1: Telomere repeat-binding factor 1
TRF2: Telomere repeat-binding factor 2
POT1: Protection of telomeres
TIN2: TRF1-interacting nuclear factor 2
PKA: Protein kinase A
GnRH: Gonadotropin releasing hormone
EGF: Epidermal growth factor
HER2: Human epithelial growth factor 2
FGFs: Fibroblast growth factors
TGF-β: Transforming growth factor-β
IGF-I: Insulin-like growth factor 1
DIZ-3: Dimeric aryl-substituted imidazole
ncRNAs: Non-coding RNAs
miRNAs: MicroRNAs
TERC: Telomerase RNA component
TERRA: Telomere repeat-containing RNA
